# Global trends of pyrolysis research: a bibliometric analysis

**DOI:** 10.1007/s11356-023-31186-0

**Published:** 2023-11-30

**Authors:** Alejandro Márquez, Isabel Ortiz, José María Sánchez-Hervás, María Concepción Monte, Carlos Negro, Ángeles Blanco

**Affiliations:** 1 Unit for Sustainable Thermochemical Valorization, CIEMAT, Av. Complutense, 40, 28040 Madrid, Spain; 2 Department of Chemical Engineering and Materials, University Complutense of Madrid, Av. Complutense s/n, 28040 Madrid, Spain

**Keywords:** Pyrolysis, Bibliometric review, Waste valorization, Research trends, Thermochemical valorization

## Abstract

Pyrolysis has become an interesting waste valorization method leading to an increasing number of research studies in this field in the last decade. The present study aims to provide a comprehensive knowledge map of scientific production in pyrolysis, discuss the current state of research, and identify the main research hotspots and trends in recent years. The systematic review, supported by analysis of countries and institutions, keyword co-occurrence analysis, analysis of keyword trends, journal analysis, and article impact, was carried out on 6234 journal articles from the Science Citation Index Expanded database of the Web of Science Core Collection. As a result, four main research hotspots were identified: 1) characterization techniques and pyrolysis kinetic models, 2) biochar production and its main applications, 3) bio-oil production and catalytic pyrolysis, and 4) co-pyrolysis, which has become a consolidated research hotspot since 2018. Additionally, the main challenges and opportunities for future research have been identified, such as 1) the development of multi-step kinetic models for studying complex wastes, 2) the integration of biochar into other valorization processes, such as anaerobic digestion, and 3) the development of catalytic hydropyrolysis for the valorization of organic waste. This bibliometric analysis provides a visualization of the current context and future trends in pyrolysis, facilitating future collaborative research and knowledge exchange.

## Introduction

Pyrolysis consists of heating the biomass or waste residues at typically between 300 and 700 ºC in the absence of oxygen. Three different products are produced during pyrolysis: bio-oil (condensable liquid fraction), pyrolysis gas (non-condensable gases), and biochar (the solid fraction) (Demirbas & Arin 2002; Olszewski et al. 2019). Pyrolysis has gained attention in the last years due to its high efficiency and flexibility in generating a combination of liquid, gaseous, and solid products, which can serve as precursors for high value-added products such as biofuel, adsorbents, or fertilizers. Pyrolysis allows a wide range of feedstock, but the chemical properties and heterogeneity of the feed have a major influence on the yield and quality of the products obtained (Foong et al. 2021). Pyrolysis can be used to process a wide variety of waste materials, including biomass, municipal solid waste (MSW), sewage sludge, plastic waste, and waste cooking oils which makes it a versatile waste management technology (Jahirul et al. 2012). Technological advancement plays a crucial role in achieving the sustainable development goals set by the United Nations (Lim 2022). As a result, pyrolysis has received significant interest as a waste valorization technology, since it contributes to circular economy.

Pyrolysis has been explored in literature from different approaches, such as catalytic pyrolysis of plastic waste (Peng et al. 2022), modeling approaches to waste biomass (Kaczor et al. 2020), microwave-assisted catalytic pyrolysis of biomass (Ren et al. 2022), physicochemical properties of biochar (Ghodake et al. 2021), the synergetic effect of biomass and plastic co-pyrolysis (Engamba Esso et al. 2022), and E-waste pyrolysis and its sustainability (Andooz et al. 2022). Considering this aim, a bibliometric analysis, utilizing algorithms, mathematics, and statistics, is the most appropriate approach to analyze large amounts of data, to identify hidden patterns during literature reviews (Kraus et al. 2022).

Some bibliometric analyses have been already carried out on pyrolysis but they are also limited to specific fields, such as reactors (fluidized bed, hot balls, microwave, plasma, and laser) (Spreafico et al. 2021), plastic pyrolysis (Armenise et al. 2021), or agricultural use of biochar in Brasil (Arias et al. 2023), without taking into consideration all the approaches of pyrolysis studies such as pyrolysis products, operating conditions, or feedstock. These reviews do not show the state of the art encompassing the majority of pyrolysis research fields. Therefore, there is a fragmentation of knowledge that motivates the present work.

To fill this gap, a bibliometric study that englobes all pyrolysis aspects is necessary to consolidate knowledge and comprehend the current state of the art and to identify research gaps, opportunities, and promising directions for future studies (Paul et al. 2021). The main purpose is to facilitate the process for researchers to analyze the latest trends in pyrolysis technology, by looking at the evolution of keywords, the emergence of new subfields, and identifying the popularity of different research topics over time. The present study consists of a bibliometric analysis of 6234 articles about pyrolysis technology published from 2017 to 2022 filling a scientific gap in the pyrolysis field. The systematic science mapping review performed using VOSviewer software, included countries and institutions analysis, keyword co-occurrence analysis, analysis of keyword trends over the past years, journal analysis, and article impact.

## Materials and method

### Data collection and research process

In this research, the data collection is based on PRISMA statement (Liberati et al. 2009; Ranjbari et al. 2022a, 2022b). Data were retrieved on August 26, 2023, from the Science Citation Index Expanded (SCI-EXPANDED) of Clarivate Analytics Web of Science Core Collection. Pyrolysis is a global term, highly used in other scientific areas such as medicine, geography, optics, or drug delivery. Therefore, searching for the keyword “pyrolysis” on SCI-EXPANDED may return results that are unrelated to the field. Given focus of this research, several keywords addressing the pyrolysis process and its combinations were tested, including as main keywords “pyrolysis,” “torrefaction,” “bio-oil,” and “biochar.” To improve the accuracy of bibliometric studies, Usman and Ho (Usman and Ho 2020) suggested the “front-page” filtering system. The “front page” is based on the research of the keywords under the following criteria: the title (TI), the abstract (AB), author keywords (AK), and keyword plus (KP). As a result, the following search string was stablished to capture all potential articles related to pyrolysis: “pyrolysis” OR “pyrolytic” OR “pyrolyzed” OR “torrefaction” OR “bio-char” OR “bio-chars” OR “biochar” OR “biochars” OR “char” OR “chars” OR “bio-oil” OR “bio-oils” OR “bio oil” OR “bio-oils.” The time range was set from January 1, 2017, to December 31, 2022. The results were limited to only peer-reviewed articles and English-language. As a result, 7214 documents were obtained. In order to guarantee the quality of the sample under study and ensure a reliable analysis, the remaining articles were reviewed based on their titles and abstracts to exclude any irrelevant ones from the analysis. Consequently, 980 articles were excluded, leaving 6234 articles for the bibliometric analysis. In Table 1, a summary of the search string detail is presented.

**Table 1:** Summary of the data collection for the bibliometric analysis

Year	2017	2018	2019	2020	2021	2022	Total
First result	747	946	1109	1317	1511	1584	7214
Screening stage	150	183	156	158	156	177	980
Final sample	597	763	953	1159	1355	1407	6234
Database	Web of Science: SCI-EXPANDED						
Time range	January 1, 2017, to December 31, 2022						
Searched in	Title, abstract, Author keyword, Keyword Plus						
WoS code	“pyrolysis” OR “pyrolytic” OR “pyrolyzed” OR “torrefaction” OR “bio-char” OR “bio-chars” OR “biochar” OR “biochars” OR “char” OR “chars” OR “bio-oil” OR “bio-oils” OR “bio oil” OR “bio oils”						

During data collection was essential to export the articles in a file format that enabling the entire content to be analyzed in VOSviewer. The established file format was tab-delimited (Win) as it preserves all the cited content and references thereby enables VOSviewer to visualize and match additional information. Data were grouped by year of publication, and Excel 2016 was used for data processing analysis to determine international collaboration articles.

### Bibliometric analysis

There are two main types of bibliometric analysis: (1) performance analysis and (2) science mapping. The first aims to determine the contribution of research constituents, either through prominent measures such as the number of publications per year or through impact and influence indicators such as citations and the *h*-index. On the other hand, the science mapping technique is based on the study of relationships among different research constituents (Donthu et al. 2021). In order to conduct a bibliometric analysis, the use of software for data processing is required. Compared with other bibliometric software (Van Eck and Waltman 2013), VOSviewer offers data and text mining functionalities that involve the identification of unknown terms extracted from a body of scientific literature (Hong et al. 2019), the visualization and exploration effect of VOSviewer maps are complete and the methodology is relatively straight forward (Zhou et al. 2021). VOSviewer software can process a large number of documents from a wide range of analytical perspectives. VOSviewer bibliometric analysis is based on heat maps and network density maps that offer data and text mining capabilities (Shah et al. 2019). VOSviewer allows setting several parameters for bibliometric analysis (van Eck and Waltman 2010). In this study, the research constituents studied are the follow: countries, institutions, author keywords, journals, and articles.

The articles were grouped by year and then imported into VOSviewer 1.6.18. Nodes and links together constitute a network and map based on network data. The size of the items on the map represents their relevance. The bigger the size, the more important it is on the field. Also, the more centered the circle is, the larger the connection with the topic. The line linking two nodes represents the number of times the two items appear to occur in the same article. As a result, the thicker the line, the more it is displayed in a document (Van Eck and Waltman 2013; Zhou et al. 2021). Different keywords with the same meaning were combined and treated as one according to previous studies (Mela et al. 1999), articles without addresses were removed, and articles from England, North Ireland, Scotland, and Wales were grouped under the United Kingdom (Ho et al. 2021).

In order to include both theory and practical issues in this study, two perspectives have been followed: 1) theoretical contribution in science mapping to identify the main clusters and thus present the state of the field and track its evolution, thereby enhancing the understanding of the development of pyrolysis technology (Mukherjee et al. 2022) and 2) practical contribution by evaluating the productivity and impact of the parameters studied (Lim et al. 2022).

## Results and discussion

### Pyrolysis article published trend

**Fig. 1:**
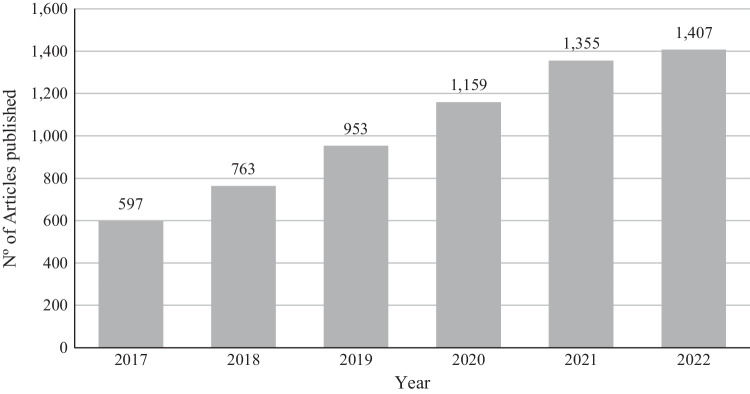
Evolution of the number of articles published about pyrolysis per year, from 2017 to 2022

Although pyrolysis is a well-known process, it has received considerable attention in recent times, becoming a hotspot as a substitute technology for non-renewable fossil fuels, waste valorization, and the promotion of a circular economy. In the previous ten years, the number of articles published on pyrolysis has grown dramatically, and focus on the last six years, the number of articles has doubled from 597 in 2017, to 1407 in 2022 (Fig. 1). This trend reveals a continuous interest of researchers in the field. However, it seems that growth is stabilizing, as the increase in 2022 compared to 2021 is not as steep.

### Country and organization analysis

Over the past six years, 6234 pyrolysis papers have been published by 118 countries. Table 2 displays the contribution evolution from the top 15 countries in pyrolysis articles production from 2017 to 2022. China dominates the period under study, contributing 56% of the published pyrolysis articles. USA and India placed second and third, with 12% and 5.4%, respectively. All the countries in the top 15 have increased the number of articles published on pyrolysis in recent years. Table 2 also shows the number of collaborative articles and their percentage of the total. It is worth noting that China has the highest number of collaborative articles, with 968 articles published between 2017 and 2022, representing approximately 35% of the total. Pakistan (84%), Germany (83%), and the United Kingdom (82%) are among the top 15 countries with the highest percentage of collaborative articles published. This pattern has persisted throughout time. China (35%), Brazil (39%), and India (44%) have the lowest percentages of collaborative papers of the top fifteen.

**Table 2:** Evolution of Top 15 productive countries in pyrolysis research from 2017 to 2022

Country	2017–2022			2017			2018			2019			2020			2021			2022		
	Rank	TA (%)	CA (%)	Rank	TA (%)	CA (%)	Rank	TA (%)	CA (%)	Rank	TA (%)	CA (%)	Rank	TA (%)	CA (%)	Rank	TA (%)	CA (%)	Rank	TA (%)	CA (%)
China	1	2730 (56)	968 (35)	1	195 (33)	93 (48)	1	323 (42)	162 (50)	1	396 (42)	127 (32)	1	510 (44)	186 (36)	1	615 (45)	194 (25)	1	691 (49)	206 (30)
USA	2	670 (12)	475 (71)	2	96 (16)	68 (71)	2	99 (13)	84 (85)	2	121 (13)	73 (60)	2	127 (11)	94 (74)	2	121 (8.9)	81 (67)	2	106 (7.5)	75 (71)
India	3	560 (5.4)	249 (44)	3	37 (6.2)	23 (62)	6	49 (6.4)	39 (80)	3	66 (6.9)	18 (27)	4	124 (11)	36 (29)	3	128 (9.4)	54 (19)	3	156 (11)	79 (51)
South Korea	4	300 (4.7)	191 (64)	8	23 (3.9)	15 (65)	4	34 (4.5)	28 (82)	6	59 (6.2)	32 (54)	3	53 (4.6)	31 (58)	6	76 (5.6)	47 (36)	4	55 (3.9)	38 (69)
Australia	5	284 (4.5)	227 (80)	5	33 (5.5)	24 (73)	3	35 (4.6)	32 (91)	4	38 (4.0)	27 (71)	5	47 (4.1)	37 (79)	4	73 (5.4)	59 (68)	7	58 (4.1)	48 (83)
United Kingdom	6	283 (3.8)	232 (82)	6	31 (5.2)	23 (74)	5	42 (5.5)	36 (86)	6	47 (4.9)	38 (81)	7	52 (4.5)	46 (88)	5	51 (3.8)	43 (79)	6	60 (4.3)	46 (77)
Malaysia	7	264 (3.7)	206 (78)	4	34 (5.7)	25 (74)	7	34 (4.5)	29 (85)	5	35 (3.7)	21 (60)	12	32 (2.8)	26 (81)	8	77 (5.7)	63 (56)	5	52 (3.7)	42 (81)
Brazil	8	236 (3.3)	93 (39)	11	18 (3.0)	8 (44)	9	27 (3.5)	18 (67)	10	39 (4.1)	14 (36)	9	44 (3.8)	18 (41)	7	53 (3.9)	19 (22)	10	55 (3.9)	16 (29)
Canada	9	231 (3.1)	155 (67)	10	21 (3.5)	17 (81)	12	25 (3.3)	20 (80)	8	34 (3.6)	17 (50)	5	43 (3.7)	29 (67)	14	53 (3.9)	32 (93)	14	55 (3.9)	40 (73)
Spain	10	217 (3)	133 (61)	6	31 (5.2)	22 (71)	8	36 (4.7)	27 (75)	9	34 (3.6)	16 (47)	11	30 (2.6)	16 (53)	10	42 (3.1)	18 (68)	15	44 (3.1)	34 (77)
Pakistan	11	202 (2.7)	169 (84)	16	15 (2.5)	11 (73)	10	23 (3.0)	21 (91)	10	31 (3.3)	25 (81)	14	29 (2.5)	25 (86)	11	52 (3.8)	42 (79)	9	52 (3.7)	45 (87)
Germany	12	190 (2.7)	157 (83)	11	18 (3.0)	14 (78)	14	21 (2.8)	19 (90)	10	35 (3.7)	27 (77)	14	35 (3.0)	30 (86)	9	37 (2.7)	28 (78)	10	44 (3.1)	39 (89)
France	13	173 (2.5)	137 (79)	13	17 (2.8)	15 (88)	17	19 (2.5)	14 (74)	14	28 (2.9)	24 (86)	9	32 (2.8)	29 (91)	11	46 (3.4)	33 (28)	10	31 (2.2)	22 (71)
Italy	14	150 (2.4)	93 (62)	8	23 (3.9)	17 (74)	13	24 (3.1)	16 (67)	19	23 (2.4)	14 (61)	13	23 (2.0)	11 (48)	14	32 (2.4)	23 (26)	16	25 (1.8)	12 (48)
Turkey	15	150 (2.4)	63 (42)	15	16 (2.7)	10 (63)	10	9 (1.2)	4 (44)	10	26 (2.7)	9 (35)	16	23 (2.0)	8 (35)	16	47 (3.5)	24 (56)	20	29 (2.1)	8 (28)

TA: Total articles; CA: Articles in international collaboration

**Fig. 2:**
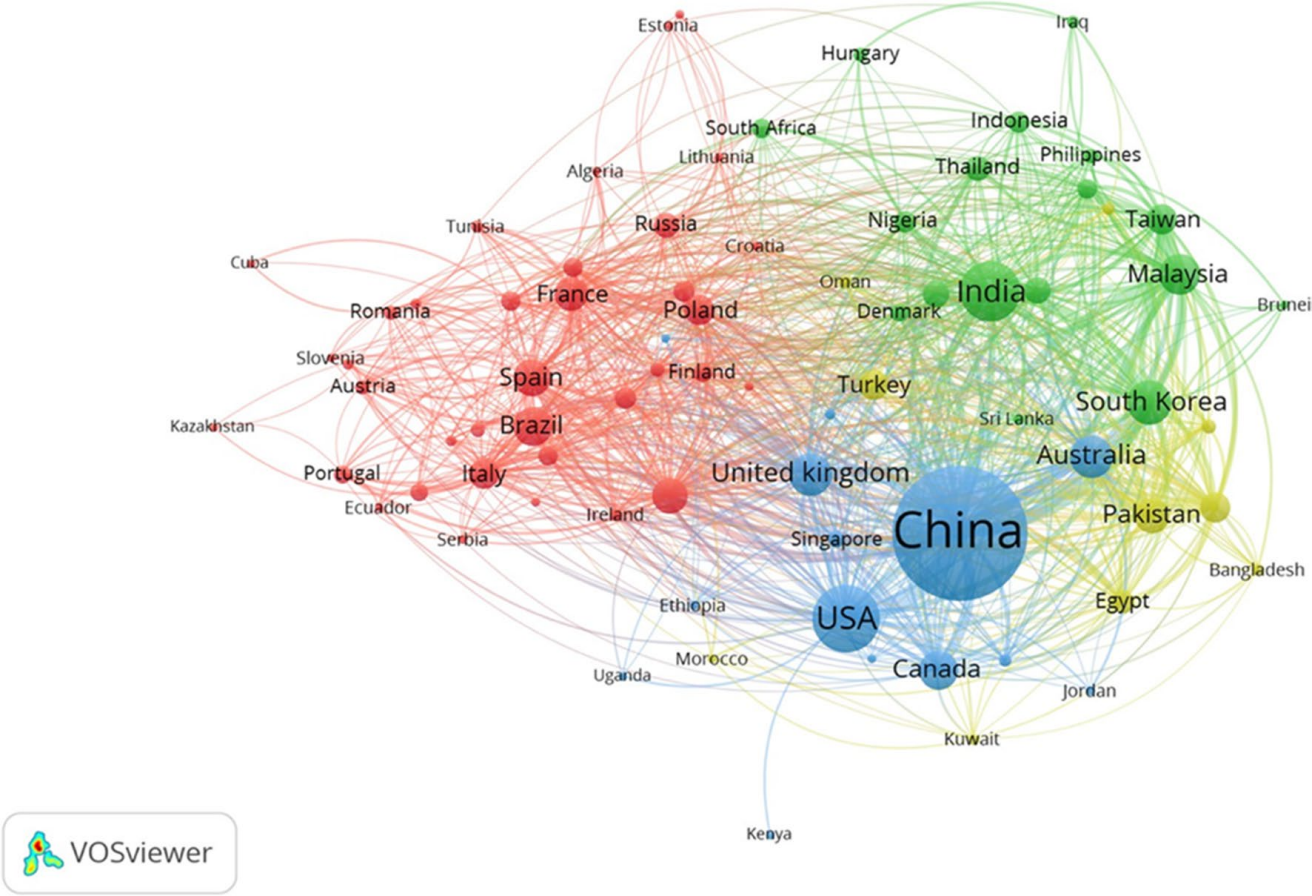
Country co-authorship of pyrolysis research. Blue cluster: most relevant countries; red cluster: European and Latin-American countries; green cluster: Asian countries, yellow cluster: Middle Eastern countries

Figure 2 illustrates international collaboration among countries in pyrolysis research. There are four clusters of collaborative activity. The blue cluster represents the countries with the highest levels of international cooperation and production, with USA at the top with 61 links between different countries, followed by China 60, and Germany with 58. China emerges as the highest collaborator with 968 articles, including papers with the USA (256 articles), the UK (100 articles), and Australia (109 articles). The red cluster primarily represents nations in Europe and Latin America. Germany and Spain stand out in this cluster with cooperation with 58 and 53 countries each, while France, Italy, and Brazil have 49, 44, and 29, respectively. The green cluster primarily represents Asian countries. India, with 51 collaborations, Malaysia, with 42, and South Korea, with 38 are among the nations with the most partnerships. Finally, the last cluster represents countries from the Middle East and neighboring countries (colored in yellow). Pakistan and Saudi Arabia stand out in this cluster, cooperating with 45 and 44 different nations, respectively.

A total of 4325 different organizations have contributed to the publication of 6234 articles. The top ten most published organizations and their evolution over the last six years are shown in Table 3. Eight of the ten institutions with the highest productivity in the field are from China, one from Taiwan (National Cheng Kung University), and one from the United States (University of Minnesota). The Chinese Academy of Science is the most productive institution in pyrolysis, with 263 publications between 2017 and 2022, making up 4.2% of all articles. The University of Minnesota has the most articles on international collaboration on ratio (74), despite ranking tenth in productivity. Between 2017 and 2022, 96% of the pyrolysis articles published by the University of Minnesota were in collaboration with international institutions.

**Table 3:** Top ten productive organization on pyrolysis research from 2017 to 2022

Institution	2017–022			2017			2018			2019			2020			2021			2022		
	Rank	TA (%)	CA (%)	Rank	TA (%)	CA (%)	Rank	TA (%)	CA (%)	Rank	TA (%)	CA (%)	Rank	TA (%)	CA (%)	Rank	TA (%)	CA (%)	Rank	TA (%)	CA (%)
Chinese Academy of Science	1	263 (4.2)	104 (40)	1	26 (4.4)	13 (50)	1	39 (5.1)	17 (44)	1	36 (3.8)	19 (53)	1	51 (4.4)	17 (33)	1	59 (4.4)	22 (37)	1	52 (3.7)	16 (31)
Huazhong University Science & Technology	2	147 (2.4)	53 (36)	2	15 (2.5)	4 (27)	2	17 (2.2)	4 (24)	2	23 (2.4)	10 (43)	2	28 (2.4)	11 (39)	2	33 (2.4)	14 (42)	2	31 (2.2)	10 (32)
Shanghai Jiao Tong University	3	107 (1.7)	49 (46)	9	7 (1.2)	0 (0)	5	15 (2)	10 (67)	6	17 (1.8)	10 (59)	8	13 (1.1)	6 (46)	4	29 (2.1)	14 (48)	5	26 (1.8)	9 (35)
Zhejiang University	4	101 (1.6)	25 (25)	9	7 (1.2)	3 (43)	7	12 (1.6)	3 (25)	5	18 (1.9)	2 (11)	27	10 (0.9)	1 (10)	5	27 (2)	11 (41)	4	27 (1.9)	5 (19)
University Science & Technology China	5	100 (1.6)	29 (29)	5	8 (1.3)	3 (38)	6	13 (1.7)	7 (54)	8	14 (1.5)	3 (21)	5	14 (1.2)	3 (21)	3	30 (2.2)	12 (40)	7	21 (1.5)	1 (4.8)
Nanjing Forestry University	6	93 (1.5)	46 (49)	5	8 (1.3)	3 (38)	9	10 (1.3)	6 (60)	23	8 (0.8)	4 (50)	3	22 (1.9)	12 (55)	19	14 (1)	8 (57)	2	31 (2.2)	13 (42)
Southeast University	7	86 (1.4)	32 (37)	3	14 (2.3)	9 (64)	2	17 (2.2)	9 (53)	12	11 (1.2)	7 (64)	20	11 (0.9)	3 (27)	13	16 (1.2)	2 (13)	11	17 (1.2)	2 (12)
National Cheng Kung University	8	78 (1.3)	69 (90)	9	7 (1.2)	6 (86)	24	6 (0.8)	5 (83)	12	11 (1.2)	10 (91)	11	12 (1)	11 (92)	7	26 (1.9)	24 (92)	12	16 (1.1)	13 (81)
University Minnesota	9	77 (1.2)	74 (96)	5	8 (1.3)	8 (100)	2	17 (2.2)	17 (100)	14	10 (1)	9 (90)	4	15 (1.3)	14 (93)	23	13 (1)	13 (100)	15	14 (1)	13 (93)
Tianjin University	10	75 (1.2)	16 (21)	21	3 (0.5)	1 (33)	8	11 (1.4)	7 (64)	35	7 (0.7)	1 (14)	34	6 (0.5)	2 (33)	5	27 (2)	3 (11)	7	21 (1.5)	2 (10)

*TA* total articles, *CA* articles in international collaboration

### Keyword analysis

**Fig. 3:**
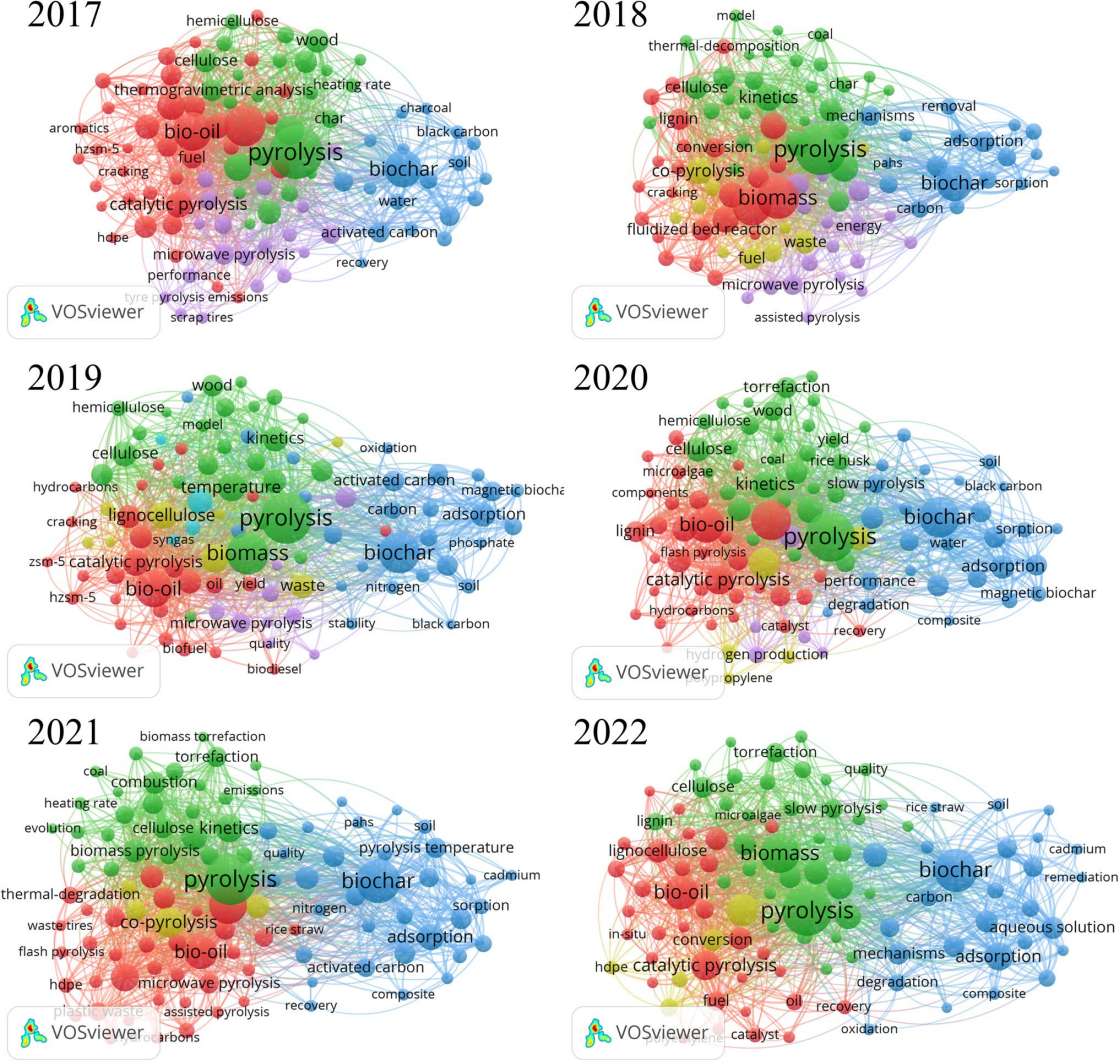
Keyword co-occurrence network related to pyrolysis from 2017 to 2022. Red cluster: research hotspot 1: characterization and pyrolysis models; blue cluster: Research hotspot 2: biochar; green cluster: research hotspot 3: bio-oil; yellow cluster: research hotspot 4: co-pyrolysis

To identify potential new pyrolysis topics and hotspots, an author-keyword co-occurrence analysis is performed for each year. The frequency with which two keywords co-occur provides insight into the strength of their relationship within a research theme. Figure 3 provides a clear thematic delimitation that makes it possible to identify the different lines of pyrolysis research hotspots. Within the context of scientific mapping, “sensing” involves comprehending the connections among topics within a cluster and the formulation of a central theme that encompasses these topics (Lim and Kumar 2023). The result of the bibliometric analysis categorized by year validates the consistency of VOSviewer software method. The co-occurrence keyword analysis map of pyrolysis reveals the different research hotspots.

#### Research hotspot 1: characterization and pyrolysis models

The first research hotspot, indicated by the red cluster, focuses on characterizing feedstock using various techniques and determining key kinetic parameters, including activation energy, pre-exponential factor, and reaction model*.* Pyrolysis is the central node of this cluster and includes terms, such as *reaction mechanism, kinetics, parameters, activation energy, thermal decomposition, and combustion.* Pyrolysis is considered a complex mechanism involving several reactions. Thus, kinetics analysis of the feedstock plays a significant role to understand the pyrolysis process. A solid understanding of pyrolysis reaction mechanism and kinetics parameters provides valuable data and guidance for designing pyrolysis reactors. Furthermore, developing pyrolysis models for simulation is increasingly important from a process optimization perspective. However, this analysis is required for each feedstock due to its significant influence on the reaction mechanism.

To determine the overall pyrolysis reaction mechanism, sample characterization is required. A vast literature is available on these characterization techniques, which are also present in this cluster. Thermogravimetric analysis (TGA) is one of the most important techniques to obtain solid data to perform a kinetics analysis. TGA is a technique used to measure changes in the weight of a sample as a function of temperature, typically under controlled heating conditions. TGA-MS can identify chemical species in the gas phase at various reaction stages, elucidating the thermal degradation mechanism. This data can be employed to understand the chemical pathways involved in the thermal degradation of the sample and how they are affected by changes in temperature and other conditions. Other techniques that are widely used in characterizing the feedstock and pyrolysis products are Fourier-transform infrared spectroscopy (*FTIR*) (De Jong et al. 2007; Fu et al. 2010) or Py-GC/MS (Lu et al. 2011; Wang et al. 2012; Zhang et al. 2015). FTIR is a non-destructive optical technique that has been successful to identify the major volatile products released during pyrolysis (Xu et al. 2018). FTIR can also be used in situ during pyrolysis, where the sample is placed in the infrared beam, and it is heated under controlled conditions, by using this method, FTIR provides information about the chemical compounds that are formed and evolved during the thermal degradation process. FTIR can also be used to study the solid residue that is produced after pyrolysis, this technique can provide information about the chemical composition of the residue and how it changes as the temperature increases. Py-GC/MS provides a qualitative and semi-quantitative analysis of pyrolysis products under different conditions. To achieve more precise studies on thermal decomposition, the coupling of different techniques has been successfully demonstrated. For instance, the combination of TGA-FTIR, TGA-MS, or FTIR-MS (Singh et al. 2012) stands out. The combination of these techniques enables a comprehensive study of the pyrolysis kinetics, product composition, and identification.

The kinetic parameters of a pyrolysis process, such as the activation energy and the reaction rate constant, can be obtained using TGA by analyzing the weight loss data of a sample as a function of temperature (Nisar et al. 2019). Isoconversional methods are the most commonly used approach for kinetic analysis in pyrolysis processes. The most widely used models are the Kissinger and Flynn–Wall–Ozawa models, and researchers often use both methods to compare energy activation. However, this technique does not yield additional results, making the differences in activation energy values obtained by these methods negligible. Another way to obtain these kinetic parameters is to use the so-called ‘model-fitting’ approach. This approach involves fitting the weight loss data into a mathematical model that describes the kinetics of the thermal degradation process (Muravyev and Vyazovkin 2022).

#### Research hotspot 2: biochar

The blue cluster represents studies on biochar, the solid carbon product obtained through pyrolysis. This cluster includes biochar´s main applications, the residues most studied to produce it, and the technologies that enhance its yield. The development of new and more efficient pyrolysis technologies is a crucial aspect of biochar research. Researchers are investigating various production methods, including *slow pyrolysis* (Anca-Couce et al. 2017) and the novel *microwave pyrolysis* (Foong et al. 2020) to optimize the yield and quality of biochar and minimize energy inputs and emissions.

In general, biochar is considered a porous material with a high surface area and abundant surface functional groups (Li et al. 2020). The physicochemical properties of biochar depend significantly on the feedstock's nature and the conditions of the pyrolysis process. In this cluster are included several wastes that have been highly studied during the last years for biochar production, such as *sewage sludge*, *municipal solid waste* (MSW), or *rice husk (*Chen et al. 2014a, 2014b; Song et al. 2014; Wang et al. 2017a; Xia et al. 2020*)*. Due to the physicochemical properties and texture of biochar, it can be used in a wide range of applications. Some of the main biochar applications are represented in this cluster, including *adsorption*. Biochar’s abundant surface area, porosity, and sorption capacity make it suitable for removing wastewater pollutants, including heavy metals, organic contaminants, nitrogen, and phosphorous compounds (Lonappan et al. 2016; Shakoor et al. 2020; Wang and Wang 2019; Wang et al. 2020). Biochar is considered a new environmentally-friendly carbon material for CO_2_ capture or pollutant removal (Tan et al. 2017). Activated carbon, another carbonaceous material, shares characteristics such as a large specific surface, extraordinary porosity, and excellent surface activity and is relevant to this cluster. According to the literature, the term activated carbon encompasses activated biochar; therefore, it is considered as a precursor for activated carbon production. Thus, activated biochar from renewable materials has the potential to be a new low-cost and environmentally-friendly carbon material (Tan et al. 2017). In addition to serving as a precursor for active carbon and an adsorbent for pollutants, biochar is also utilized in sustainable agriculture, catalytic reaction, CO_2_ capture, energy production, and lithium and sodium-ion batteries (Wang and Wang 2019). The potential applications of biochar in various fields, including agriculture, forestry, waste management, and energy production, are also being explored (El-Naggar et al. 2019).

#### Research hotspot 3: bio-oil

The green cluster corresponds to bio-oil production field. This cluster refers to the investigations on the liquid fraction produced during pyrolysis, including operating conditions that increase its yield, upgrading processes, and its final application. Fast pyrolysis is the most efficient thermochemical process to transform a feedstock into bio-oil. During fast pyrolysis, the feedstock is rapidly heated (> 100 °C) to moderate temperature between 500 and 700 °C and short residence time (< 2 s) (Bridgwater 2018; Khosravanipour Mostafazadeh et al. 2018). Operational conditions, such as particle size, heating rate, reactor type, temperature, residence time, and feedstock characteristics, influence both the yield and properties of bio-oil. For instance, typical biomass fast pyrolysis produces around 60–70 wt. % bio-oil yields, while plastic pyrolysis can reach up to 90% wt.%. Pyrolysis bio-oil is a complex mixture that contains various chemicals: organic acids, ketones, alcohols, phenols, furans, water, sulfur compounds, etc. Oxygenated organic compounds are produced during the decomposition of the biomass major components: cellulose, hemicellulose, and lignin. Oxygenated organic compounds presence reduces the heating value in bio-oil, increases corrosiveness, and increases polymerization during storage (Isahak et al. 2012). Therefore, an upgrading process for removing impurities and improving the heating values is required. Catalytic pyrolysis improves bio-oil yield and quality, and increases the reaction selectivity, reducing the number of oxygenated compounds and water content. In addition, catalytic pyrolysis can reduce waste and greenhouse gas emissions, making it a desirable option for waste management. Keyword co-occurrence analysis shows some of the major catalysts used during the last years, zeolites (HZMS-5, ZSM-5), metal oxide, and minerals. A major drawback of catalytic pyrolysis is catalyst deactivation, which can result from coking, poisoning, and sintering. Deactivation in catalytic pyrolysis can be categorized into reversible and irreversible deactivation. Reversible deactivation occurs when the coke is deposited on the external surface of the catalyst and can be removed by thermal treatment at moderate temperatures (400–600 °C) in an oxygen atmosphere. On the other hand, irreversible deactivation is caused by the poisoning of the acid sites of the catalyst (Zhang et al. 2018). The outlook for catalytic pyrolysis is promising, as catalytic pyrolysis technology has the potential to play a significant role in the transition to a more sustainable and low-carbon energy system. The development of new catalysts and reactors, as well as the optimization of reaction conditions, will likely lead to further technological advancements.ñ

#### Research hotspot 4: co-pyrolysis

Co-pyrolysis is a process in which two or more materials are pyrolyzed, generally lignocellulosic biomass feedstock, and hydrogen-rich materials such as *plastic* or *rubber*. The most studied plastics are represented in the cluster, primarily *HDPE*, *polystyrene,* and *polypropylene*. Co-pyrolyzing plastics with other materials can result in bio-oil that is more valuable than the individual components alone. Co-pyrolysis can result in a more valuable bio-oil, as the plastic and the other material can have a synergistic effect that improves the overall product yield (Abnisa and Wan Daud 2014). Adding plastic materials increased the oil production and caloric value of the fuel, which derives from hydrocarbon polymers made of paraffins, isoparaffins, olefins, naphthenes, and aromatics (Zhang et al. 2016). Co-pyrolysis offers effective waste management by reducing the amount of plastic waste ending up in landfills or the environment and utilizes waste carbon (Wang et al. 2021). It is worth noting that the advantages of co-pyrolysis will depend on the specific conditions of the process and the materials being used. Additionally, the complexity of the process and the need for a specific equipment for its implementation should be also considered.

### Pyrolysis trends

**Fig. 4:**
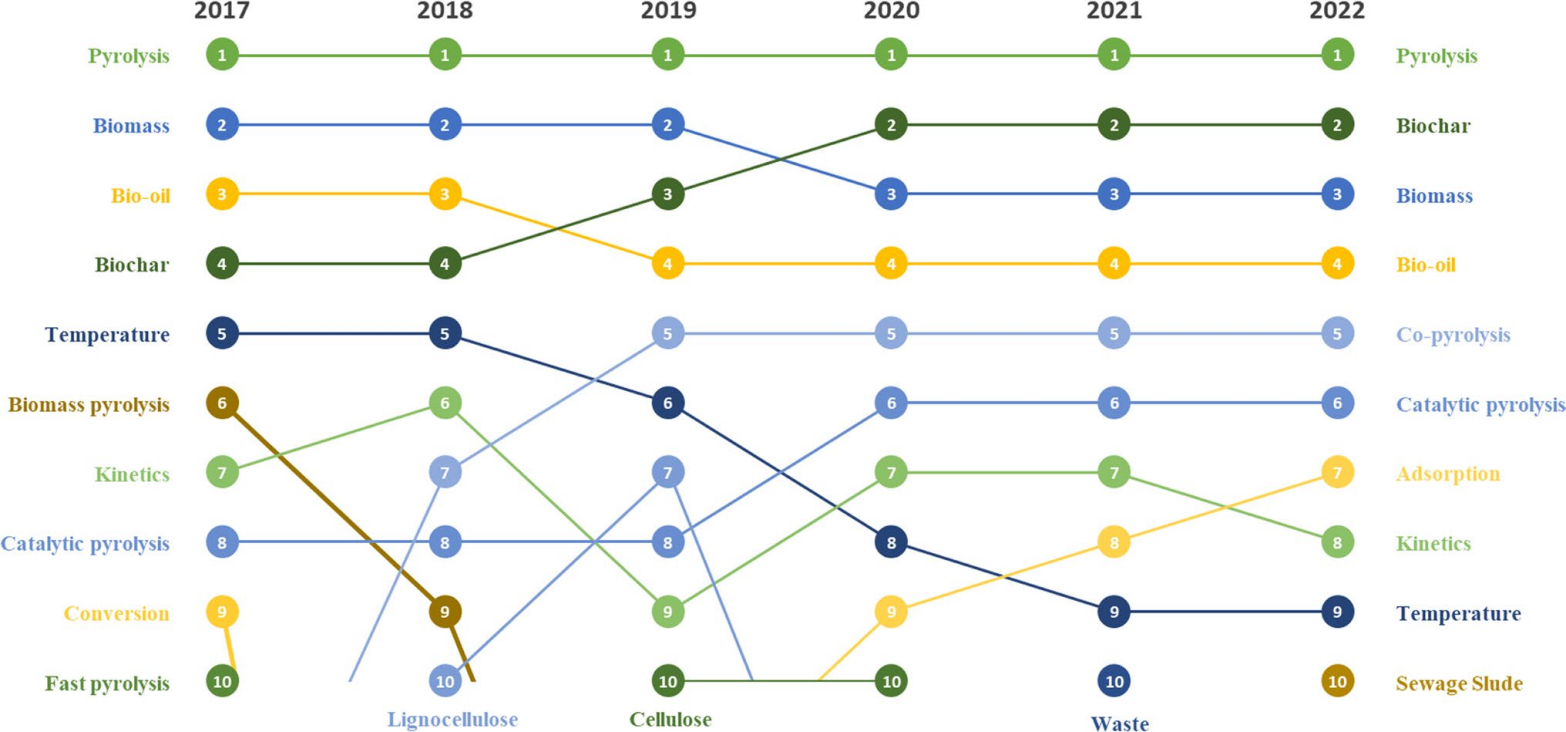
Top 10 most occurrence keywords from 2017 to 2022 on pyrolysis research

Figure 4 displays the top ten author keywords from 2017 to 2022, providing a trend analysis of pyrolysis-related hotspots. This research is based on the repetition and annual ranking of author keywords. As expected, ‘pyrolysis’ is the most often used word, due to its centrality in the analysis. ‘Temperature’ and ‘kinetics,’ keywords related to research hotspot 1 (characterization and pyrolysis models), ranked 5^th^ and 7^th^ in 2017 and 9^th^ and 8^th^ in 2022, respectively. This shift indicates a declining trend in articles related to the development of kinetic models. Another prominent trend observed is the paradigm shift driven by the global implementation of waste management and greenhouse gas emissions regulations. These regulations may have stimulated research and development efforts in more efficient, sustainable, and regulatory-compliant pyrolysis technologies, which could be reflected in changes in the popularity of certain keywords over time. Figure 4 illustrates the significant growth and recent emergence of keywords closely associated with waste management, including ‘waste’ and ‘sewage sludge.’ Notably, the keyword ‘co-pyrolysis’ has surged from the 14^th^ position in 2017 to 5^th^ in 2022. This phenomenon underscores a shift in scientific priorities regarding pyrolysis, positioning it as a technology capable of treating and reducing various wastes, and not only biomass. This is explained by the fact that as the usage of the term ‘co-pyrolysis’ increases, the rankings of the words ‘biomass’ and ‘biomass pyrolysis’ decline. Pyrolysis not only enables the valorization of waste in accordance with new legislations but also its products, specifically biochar, exhibit significant potential to support the transition toward a circular economy and reduce environmental impact The use of biochar as an adsorbent in wastewater treatment systems or remediation of contaminated soils can be a solution that complies with environmental regulations.. As shown in Fig. 4, 'biochar' has significantly risen in popularity, moving from 4th place in 2017 to the second most frequently used keyword in 2022. Additionally, its primary application, 'adsorption,' has also benefited, jumping from the 24^th^ position to 7^th^ in 2022.

### Journal analysis

A total of 6234 pyrolysis articles were published in 681 journals in the Science Citation Index Expanded. Table 4 lists the top 10 most productive journals in the field from 2017 to 2022. WoS assigns a scientific category to each journal. Eight of the top 10 journals are in the Fuel & Energy category, and four are in Chemical Engineering. The Journal of Analytical and Applied Pyrolysis (IF_2022_ = 6.0) leads with the highest number of pyrolysis publications (413) from 2017 to 2022, followed by Fuel (IF_2022_ = 7.4) with 408 articles and Bioresource Technology (IF_2022_ = 11.4) with 296. Bioresource Technology has the highest impact factor, followed by Journal of Cleaner Production (IF2022 = 11.1) and Energy Conversion and Management (IF2022 = 10.4). Regarding the evolution of each journal’s production, seven out of the top ten journals increased their article output from 2017 to 2022. Fuel has shown the most growth, increasing from 30 articles in 2017 to 109 in 2022. Science of the Total Environment (IF_2022_ = 9.8) has significantly increased its article production, with a 450% rise in 2022 compared to 2017. Nine out of the top ten journals listed in Table 4 are published by Elsevier, demonstrating their dominance in pyrolysis research.

**Table 4:** Top 10 most productive journals on pyrolysis articles

Journal	Category	IF2022	2017–2022		2017		2018		2019		2020		2021		2022	
			Rank	TA (%)	Rank	TA (%)	Rank	TA (%)	Rank	TA (%)	Rank	TA (%)	Rank	TA (%)	Rank	TA (%)
Journal of Analytical and Applied Pyrolysis	Chemistry, Analytical; Energy & Fuels; Engineering, Chemical	6	1	413 (6.6)	1	59 (9.9)	1	78 (10)	3	55 5.8)	3	45 (3.9)	1	92 (6.8)	2	84 (6.0)
Fuel	Energy & Fuels; Engineering, Chemical	7.4	2	408 (6.5)	5	30 5.0)	3	49 (6.4)	2	57 (6.0)	1	87 (7.5)	2	76 (5.6)	1	109 (7.7)
Bioresource Technology	Agricultural Engineering; Biotechnology & Applied Microbiology; Energy & Fuels	11.4	3	296 (4.7)	2	44 (7.4)	2	51 (6.7)	1	63 (6.6)	2	51 (4.4)	5	49 (3.6)	7	38 (2.7)
Energy	Energy & Fuels; Thermodynamics	9	4	216 (3,5)	6	22 (3.7)	5	28 (3.7)	4	41 (4.3)	9	33 (2.8)	4	53 (3.9)	5	39 (2.8)
Science of the Total Environment	Environmental Sciences	9.8	5	180 (2,9)	16	7 (1.2)	8	21 (2.8)	15	16 (1.7)	5	42 (3.6)	3	55 (4.1)	5	39 (2.8)
Journal of Cleaner Production	Engineering, Environmental; Environmental Sciences; Green & Sustainable Science & Technology	11.1	6	173 (2.8)	14	9 (1.5)	10	19 (2.5)	6	26 (2.7)	5	42 (3.6)	8	37 (2.7)	4	40 (2.8)
Energy Conversion and Management	Energy & Fuels; Mechanics; Thermodynamics	10.4	7	154 (2.5)	3	32 (5.4)	4	31 (4.1)	14	17 (1.2)	8	34 (2.9)	21	15 (1.1)	12	25 (1.8)
Biomass conversion and Biorefinery	Energy & Fuels; Engineering, Chemical	4	8	151 (2.4)	10	12 (2.0)	26	5 (0.7)	27	7 (0.7)	4	44 (3.8)	8	37 (2.7)	3	56 (4.0)
Fuel Processing Technology	Chemistry, Applied; Energy & Fuels; Engineering, Chemical	7.5	9	144 (2.3)	3	32 (5.4)	9	20 (2.6)	9	20 (2.1)	17	16 (1.4)	12	30 (2.2)	11	26 (1.8)
Renewable Energy	Energy & Fuels; Green & Sustainable Science & Technology	8.7	10	140 (2.2)	25	5 (0.8)	15	11 (1.4)	5	27 (2.8)	7	36 (3.1)	14	27 (2.0)	8	34 (2.4)

*TA* total articles

### Highly cited paper analysis

**Fig. 5:**
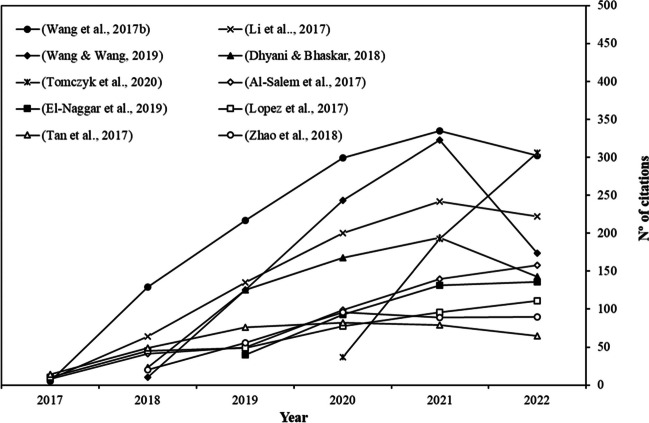
Citation evolution of the top ten pyrolysis articles from 2017 to 2022

The study of the most relevant papers with the greatest scientific impact is another important part of bibliometric analysis. Citations in an article are essential for this purpose. However, it is important to note that the number of citations is directly related to the year of publication. Therefore, we have taken into account both the citations for each year and their progression over time. In their study, Ho and Hartley (Ho and Hartley 2016) suggested that researchers should pay particular attention to recent highly impactful works. This involves considering citations from the most recent year, which in this case is 2022 (*C*_2022_). Table 5 presents the top ten pyrolysis articles with the highest number of citations from 2017 to 2022 (*C*_2017–_ _2022_). Figure 5 displays the evolution of the annual number of citations. In the period between 2017 and 2022, among the top 10 most cited articles on pyrolysis, among the top 10 most cited articles on pyrolysis from 2017 to 2022, nine are review articles, while the tenth position is held by a research article (Zhao et al. 2018). The articles in Table 5 have been categorized based on the research hotspots described in “Keywords analysis”.

**Table 5:** Top ten most cited pyrolysis articles published between 2017 and 2022

Rank	Article title	*C*_2017–2022_	*C*_2022_	Reference
1	Lignocellulosic biomass pyrolysis mechanism: A state-of-the-art review	1287	302	(Wang et al. 2017b)
2	Preparation, modification and environmental application of biochar: A review	875	174	(Wang and Wang 2019)
3	Mechanisms of metal sorption by biochars: Biochar characteristics and modifications	871	222	(Li et al. 2017)
4	A comprehensive review on the pyrolysis of lignocellulosic biomass	653	143	(Dhyani and Bhaskar 2018)
5	Biochar physicochemical properties: pyrolysis temperature and feedstock kind effects	536	306	(Tomczyk et al. 2020)
6	A review on thermal and catalytic pyrolysis of plastic solid waste (PSW)	496	158	(Al-Salem et al. 2017)
7	Biochar application to low fertility soils: A review of current status, and future prospects	400	136	(El-Naggar et al. 2019)
8	Thermochemical routes for the valorization of waste polyolefinic plastics to produce fuels and chemicals. A review	389	111	(Lopez et al. 2017)
9	Biochar as potential sustainable precursors for activated carbon production: Multiple applications in environmental protection and energy storage	365	65	(Tan et al. 2017)
10	Effect of pyrolysis temperature, heating rate, and residence time on rapeseed stem derived biochar	351	90	(Zhao et al. 2018)

*C*_*2017–2022*_ total citation from 2017 to 2022, *C*_*2022*_ total citation in 2022

Six of the 10 most cited articles are related to the production, characterization, and application of biochar (Research Hotspot 2). In their comprehensive review, Wang and Wang cover the production, modification, characterization, and environmental application of biochar. This includes its role in soil remediation, carbon sequestration, organic solid waste composting, water and wastewater decontamination, catalytic uses, and its potential as an electrode material (Wang and Wang 2019). Furthermore, the articles by Tomczyk et al. (2020) and El-Naggar et al. (2019) delve deeper into the application of biochar to enhance soil fertility and productivity. These studies conclude that there is significant potential for rehabilitating low fertility soils, especially when co-composting with organic materials, which could maximize the economic benefits of the process. Another impactful application of biochar is in the adsorption mechanisms of metals present in aqueous solutions. The metals most extensively studied include arsenic, chromium, cadmium, lead, and mercury. Although biochar holds promise for removing metals from water, the recovery of metals sorbed and the regeneration of it still present challenges that need to be addressed before its widespread acceptance in wastewater treatment (Li et al. 2017). Finally, in the article authored by Tan et al., the potential of biochar as a precursor for the production of activated carbon through physical activation and chemical activation processes is discussed. In this article, researchers could find a compilation of the primary raw materials used and the most commonly employed activation methods for the production of activated carbon. Notable applications highlighted include the removal of organic compounds from water, such as dyes or pharmaceutical substances, the capture of CO_2_ based on biochar’s surface properties, and its utilization in energy storage, as electrode materials for supercapacitors or as a porous matrix for hosting active substances in cathodes (Tan et al. 2017).

The most influential pyrolysis article between 2017 and 2022, authored by Wang et al., explores the degradation and reaction mechanisms of the three primary biomass components: cellulose, hemicellulose, and lignin. Additionally, it discusses experimental techniques such as Py-GC-MS/FID, TG-FTIR, and TG-MS, which unveil the mechanisms behind biomass pyrolysis, along with kinetic models for determining crucial kinetic parameters (Research Hotspot 1). Furthermore, the article places a special focus on the optimization of pyrolysis product distribution, especially through catalytic pyrolysis, and emphasizes the utilization of inorganic minerals, metal oxides, and zeolites as catalyst (Research Hotspot 3) (Wang et al. 2017b). The development of integrated bio-refineries with a supply of high-quality feedstock, inexpensive and simple-to-regenerate catalysts for bio-oil upgrading, reactors with high thermal efficiencies, and an energy market that adapts will be necessary for the successful and sustainable use of pyrolytic oils to replace fossil fuels.

Lastly, the articles concerning the valorization of plastic fractions through pyrolysis are compiled. Since plastic thermal pyrolysis occurs via a random scission mechanism, it typically results in a broad range of products. One of the primary challenges in plastic pyrolysis is the low thermal conductivity of plastics, which hinders efficient and uniform heating of the polymer within the reaction environment. Additionally, melted plastic tends to be sticky, leading to significant operational issues (Al-Salem et al. 2017; Lopez et al. 2017). Consequently, the proper selection and optimization of the reactor are essential. To achieve a narrower and more valuable product range, various strategies have been explored, including catalytic cracking to generate a variety of useful hydrocarbons that can potentially serve as chemical feedstocks or energy sources (Lopez et al. 2017). In these articles, both Lopez et al., and Al-Salem et al., make a special mention of the co-pyrolysis of waste plastics with other solid residues, primarily biomass (Research Hotspot 4). However, most of the studies conducted so far have been carried out in laboratory-scale reactors and operated in a batch regime, which limits their scalability. Nonetheless, co-pyrolysis of plastics with other solid wastes seems to offer a promising path for valorization due to the enhancement of the overall product qualities compared to when these materials are processed separately. This enhanced product quality is particularly evident in the case of catalytic co-pyrolysis (Al-Salem et al. 2017).

## Future recommendations: challenges and opportunities

Pyrolysis holds the potential to play a crucial role in facilitating the transition towards a circular economy by closing the loop of waste recycling as the last step of the chain. However, there are still several bottlenecks and challenges that impede the development and integration of pyrolysis technologies at large scale.

One significant concern is related to kinetic models due to the limited number of studies considering biomass as a multi-component material. As a result, there is a significant variation of over 20% in the observed activation energy during the pyrolysis process, suggesting the complexity of the multi-stage process. Furthermore, the widespread use of isoconversional models like Kissinger–Akahira–Sunose, Kissinger, or Flynn–Wall–Ozawa poses another issue, as these models often yield trivial activation energy results without significant differentiation (Vyazovkin et al. 2020). Hence, to enable the implementation of pyrolysis for biomass/waste treatment, it is crucial to develop models that consider biomass pyrolysis, as well as other residues with multiple components, as a multi-stage process. This allows for the determination of each component's contribution. Finally, the development and implementation of kinetic modeling software that promotes research transparency are crucial for advancing the field (Muravyev and Vyazovkin 2022).

The potential application of biochar is a hotspot research topic, with particular emphasis on its noteworthy role as an adsorbent. While recent studies have primarily focused on optimizing the activation process conditions, it is also crucial to assess the environmental and economic implications of producing activated carbons from biochar. Another challenge that needs attention is the investigation of interactions between biochar and the matrix, especially when dealing with complex matrices like wastewater, and how these interactions affect the adsorption capacity of biochar. Moreover, there is a need to explore and develop the integration of biochar into other valorization processes, including anaerobic digestion. This area requires dedicated research and innovation to comprehensively understand the potential benefits and optimize the synergies between biochar and anaerobic digestion processes. (Chiappero et al. 2020). Finally, the regeneration and final disposal of spent biochar need to be further explored specially when they are used for the removal of highly toxic compounds and emerging contaminants.

In the case of bio-oil, the reduction of oxygenated compounds in its final composition is a major area of research. Zeolites have been extensively studied as catalysts due to their high specific surface area and abundant active acid sites, which facilitate deoxygenation reactions. However, it is crucial not only to reduce the oxygen content but also to consider the specific method of oxygen removal. Hydrodeoxygenation reactions, which eliminate oxygen in the form of H_2_O, result in a higher H/C molar ratio compared to decarboxylation and decarbonylation reactions (Oi et al. 2016). Catalytic hydropyrolysis, involving pyrolysis under a reactive atmosphere with H_2_, presents a promising alternative for enhancing the reaction mechanisms through hydrodeoxygenation. Nevertheless, further studies, particularly focusing on catalyst deactivation, are necessary to advance in this field of research.

Although pyrolysis results in three products (biochar, bio-oil, and non-condensable gases), the research on the gas fraction is significantly less extensive. The primary reason for this disparity is that the gases are predominantly used as fuel due to the highly endothermic nature of the pyrolysis process. However, it is essential to address the potential presence of contaminants, such as H_2_S and HCl, in these gases, which requires a thorough cleaning process. This aspect deserves considerable attention for the implementation of the technology, particularly in the context of pyrolyzing complex feedstocks like municipal solid waste, as it could lead to catalyst poisoning and pose additional challenges.

## Conclusions

Pyrolysis is a well-known technology in the scientific community, and it has gained significant interest in the transition from a linear to a circular economy. An analysis of 6234 articles from the Science Citation Index Expanded was conducted to assess publication trends, the most prolific countries and organizations, journals, and research hotspots. This bibliometric analysis identifies the trends in the current pyrolysis state-of-the-art over the last 6 years, which can help researchers to anticipate the progression of the pyrolysis technology.

The number of pyrolysis papers published has more than doubled in the last six years, indicating a rising trend in scientific productivity. In the period 2017–2022, China was identified as the main country in publication, producing 56% of the pyrolysis articles, followed by the United States (12%), and India (5.4%). China, the United States, and India have the most articles in international collaboration, with 968, 475, and 249 articles, respectively. Eight of the top ten pyrolysis-producing institutions are Chinese, with the Chinese Academy of Science having the highest production at 4.2% of the total. The University of Minnesota, on the other hand, has the most international collaboration ratio, with 74 papers published between 2017 and 2022. Regarding journals, the top three publishing the most pyrolysis articles were *Journal of Analytical and Applied Pyrolysis* (IF_2022_ = 6.0) with 413 articles, *Fuel* (IF_2022_ = 7.4) with 408 articles, and *Bioresource Technology* (IF_2022_ = 11.4) with 296 articles.

Co-occurrence keyword analysis reveals four different pyrolysis research hotspots: (1) Research Hotspot 1 focuses on characterizing feedstock and understanding pyrolysis kinetics, which is crucial for reactor design. (2) Research Hotspot 2 highlights the significance of biochar production, applications, and optimization. (3) Research Hotspot 3 centers on bio-oil production, its up-grading and potential applications. (4) Research Hotspot 4 discusses co-pyrolysis, particularly combining biomass and plastics, to enhance product value. Trends in pyrolysis research include a shift towards waste management and environmental compliance to support the circular economy transition. There is a growing interest in co-pyrolysis beyond biomass, indicating a broader range of applications. Moreover, potential future research include: (I) the need for improved kinetic models, especially for multi-component feedstocks. (II) Biochar research should consider environmental and economic implications and integration with other valorization processes, especially anaerobic digestion. (III) The gas fraction in pyrolysis, particularly in complex feedstocks, deserves more attention.

## Data Availability

Not applicable.
